# The Conqueror Worm: recent advances with cholinergic anthelmintics and techniques excite research for better therapeutic drugs

**DOI:** 10.1017/S0022149X1400039X

**Published:** 2014-05-29

**Authors:** R.J. Martin, S. Puttachary, S.K. Buxton, S. Verma, A.P. Robertson

**Affiliations:** Department of Biomedical Sciences, College of Veterinary Medicine, Iowa State University, USA

## Abstract

The following account is based on a review lecture given recently at the British Society of Parasitology. We point out that nematode parasites cause very widespread infections of humans, particularly in economically underdeveloped areas where sanitation and hygiene are not adequate. In the absence of adequate clean water and effective vaccines, control and prophylaxis relies on anthelmintic drugs. Widespread use of anthelmintics to control nematode parasites of animals has given rise to the development of resistance and so there is a concern that similar problems will occur in humans if mass drug administration is continued. Recent research on the cholinergic anthelmintic drugs has renewed enthusiasm for the further development of cholinergic anthelmintics. Here we illustrate the use of three parasite nematode models, *Ascaris suum, Oesophagostomum dentatum* and *Brugia malayi,* microfluidic techniques and the *Xenopus* oocyte expression system for testing and examining the effects of cholinergic anthelmintics. We also show how the combination of derquantel, the selective nematode cholinergic antagonist and abamectin produce increased inhibition of the nicotinic acetylcholine receptors on the nematode body muscle. We are optimistic that new compounds and combinations of compounds can limit the effects of drug resistance, allowing anthelmintics to be continued to be used for effective treatment of human and animal helminth parasites.

## Introduction

It writhes!–it writhes!–with mortal pangs

The mimes become its food,

And seraphs sob at vermin fangs

In human gore imbued

(*The Conqueror Worm,*Edgar Allan Poe, 1843)

**Fig. 1:**
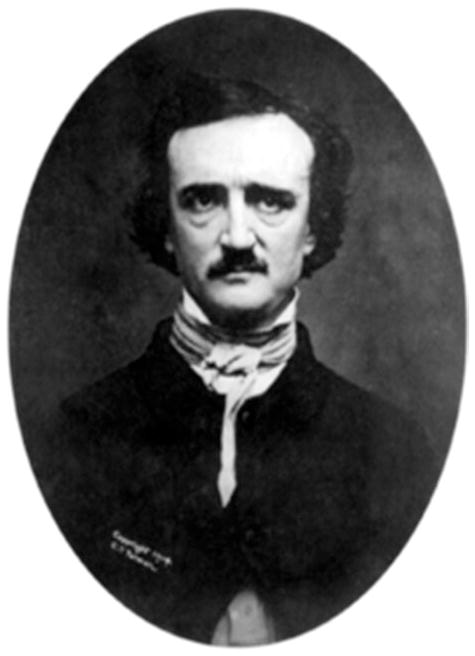
Edgar Allan Poe, the author of the poem *The Conqueror Worm*, which was first published in 1843. Daguerreotype of Poe by William S. Hartshorn (1848), Library of Congress, Prints and Photographs Division [#91796062].

We start our account with a quote from a poem, *The Conqueror Worm,* which was first published in 1843 in *Graham’s Magazine*. It is a poem by Edgar Allan Poe (fig. 1), about the inevitability of death. Did Poe know about the horrors of parasitic nematodes? Probably not, but Poe’s parents were actors in the theatre where metaphors were used to describe life’s events and tragedies. In his poem, *The Conqueror Worm*, the word ‘worm’ is a metaphor for death, and is used in the same way as many ancients used this word. The ancients saw the actions of worms in the process of death and decay. *The Conqueror Worm* is interpreted to mean that human life is a mad folly ending in horrid death. The lines, above, from the poem describe the worm devouring, in a bloody manner, humans, who are mimes or puppets, while the do-gooders (seraphs) weep to no effect. The poem is also a strong metaphor for the effects of severe nematode parasitism on humans and the sometimes ineffective actions of concerned governments. In contrast to the tone of this poem, we choose to be optimistic, knowing that treatment with drugs can defeat the conqueror worm; but we need to know the weaknesses of the ambitious conqueror and to use and develop stronger weapons. This paper presents some recent insights into some of the mechanisms of actions of cholinergic anthelmintics and illustrates the potential use of microfluidic and expression techniques that facilitated recent advances in this field.

## Nematode parasites are a major world problem

A group of diseases referred to as the neglected tropical diseases (NTDs) have an impact on significantly more people than HIV/AIDS, malaria and tuberculosis. The NTDs are mostly caused by parasitic nematodes. They may not kill, but they produce malnutrition that leads to debility, poor growth, reduced intellectual development, limb disfigurement and an increase in HIV/AIDS infection rates (Bentwich *et al.*, 2008). These diseases are associated with poverty, poor sanitation and poor education. The diseases perpetuate the poverty cycle because infected children cannot attend school, so they do not reach their full intellectual potential, and infected adults are less productive. Also important for human health is proper nutrition, which is adversely affected by the production loss caused by parasitic gastrointestinal nematodes in livestock.

We have used three parasitic nematodes that are models for NTDs and tractable to our molecular, pharmacological and electrophysiological methods. The nematodes are: (1) *Ascaris suum* (or *lumbricoides,* var. *suum*) which can produce ascariasis in humans and pigs; (2) *Brugia malayi,* which produces elephantiasis in humans; and (3) *Oesophagostomum dentatum*, which produces nodule formation and dysentery oesphagostomiasis in pigs and is a model for *Oesophagostomum bifurcum* which produces similar pathology in goats, pigs and humans in Togo and Ghana (Storey *et al.,* 2000).

**Fig. 2:**
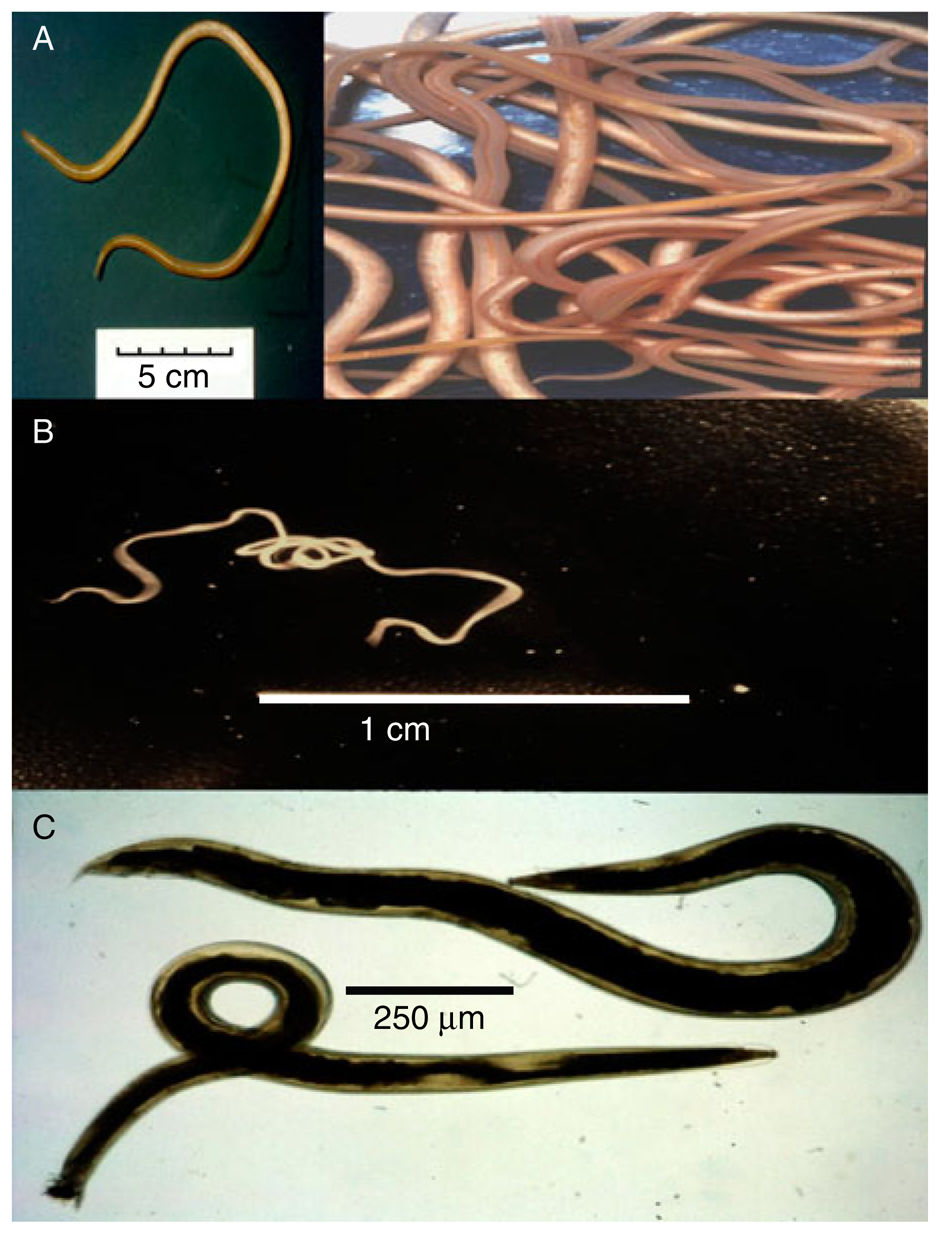
(colour online) *Ascaris, Brugia* and *Oesophagostomum* adult worms: (A) *Ascaris suum*, a single worm and scale bar (inset) and active swimming worms; (B) *Brugia malayi*, a single motile worm; (C) *Oesophagostomum dentatum*, female (top), male (bottom).

Ascariasis is the most common human worm infection and is due to the large (20 cm) parasitic nematode, *A. lumbricoides*, which lives in the gastrointestinal tract. The worm is genomically very similar to, and may represent the same species as, *A. suum* of pigs (fig. 2A) (Liu *et al.*, 2012). Ascariasis has a global prevalence of 36% (Chan *et al.*, 1994; Brooker *et al.*, 2013), producing symptoms of malnutrition, retardation of growth in children, diarrhoea, abdominal pain and death in a smaller proportion of cases (Bethony *et al.*, 2006). Infection occurs in an estimated 1.4 billion people worldwide. Infection is spread by the faecal–oral route, and involves ingestion of embryonated eggs. Parasite infections, like ascariasis, which are spread by the faecal–oral route, are referred to as soil-transmitted nematodes (STNs). Worldwide, STNs are the major cause of morbidity in schoolchildren aged 5–14 years (Hotez, 2007).

Lymphatic filariasis is another important NTD, produced by filarial nematodes like *B. malayi* (fig. 2B). These thread-like worms live in lymphatic vessels of humans for up to 6 years. Some 119 million individuals have been estimated to be afflicted with lymphatic filariasis (Michael *et al.,* 1996), and many more (1 billion people worldwide) are at risk of contracting lymphatic filariasis and related filarial diseases. Transmission is via mosquitoes that bite and pick up microfilaria, which develop inside the mosquito into infective stages over 7–21 days. The larvae enter the mouthparts of the mosquito and then enter the punctured skin of the human following feeding. Although the infection may be symptomless, in about 10% of infected individuals blockage of the lymphatic vessels causes swelling known as elephantiasis. Elephantiasis is a gross swelling of the infected tissues that leaves individuals with severe disfigurement, an inability to work and sometimes exclusion from the social group.

Oesophagostomiasis infections in humans due to *O. bifurcum* are localized in northern Togo and Ghana (Storey *et al.,* 2000). The parasites produce nodules in the large colon and sometimes more serious dysentery. We are able to maintain a very similar parasite, *Oesophagostomum dentatum,* which causes infections of pigs (fig. 2C), by relatively simple passage techniques. We also have isolates of levamisole-sensitive and levamisole-resistant *O. dentatum*.

## How can we control parasitic worms in general?

If we have a medical problem, then information on the cause of that problem allows us to limit, control and sometimes cure that problem. The more detailed and accurate our information is, the more effective the treatment is likely to be. Control of nematode parasitic disease has three focal points:

- preventative management, including sanitation and hygiene;
- vaccination;
- anthelmintic treatment.

Preventive management with effective sanitation and appropriate hygiene requires social cohesion and basic institutional and communal organization, which is available in developed economies but is not universally available. The essential components are clean water and effective sewage disposal; major advances in the control of the parasitic diseases would be possible if these were to be available and were maintained effectively. Vaccines against parasitic nematodes are very desirable but are not yet available. Trials with vaccines using two antigens – *Necator americanus* glutathione s-transferase 1 and *N. americanus* aspartic protease – are in phase 1 (Beaumier *et al.*, 2013). Vaccines against animal parasites, including lungworm (*Dictyocaulus viviparous*) and *Haemonchus contortus* (Albonico *et al.*, 1994; Knox *et al.*, 2003; Smith & Zarlenga, 2006), have been produced, but of the few parasite vaccines that have been produced commercially, nearly all are based on attenuated organisms. The task is to identify stable antigens that induce strong immunity in the field, and many candidate antigens are from the excretory substance (ES) or the gut antigens of the nematode parasite. Despite the need for improved preventive management and vaccines, for many humans and animals, drugs remain the only option for treatment and prophylaxis.

## Anthelmintics are used for treatment and prophylactic control

Over the past few years, governments of developing countries, WHO and public–private partnerships have collaborated to overcome the NTDs, but the fight has just started. In the absence of vaccines, and where sanitation and vector control is limited, control of these infections relies on regular administration of anthelmintic drugs.

For control of STN infections such as ascariasis, anthelmintic chemotherapy has focused on a small number of drugs that belong to three major groups (Martin, 1997): (1) benzimidazoles, like albendazole, bind to nematode β-tubulin and act over several days to inhibit metabolism and egg production; (2) nematode-selective nicotinic compounds (levamisole and pyrantel) act more rapidly and produce spastic paralysis; and (3) avermectins, such as ivermectin and abamectin, inhibit motility and pharyngeal pumping.

In endemic communities, the goal for control of filariasis has been to eliminate microfilaria from blood to interrupt transmission. Single-dose diethylcarbamazine with albendazole, or albendazole with ivermectin (Ottesen, 2000) is advocated in the Global Program to Eliminate Lymphatic Filariasis. These drugs do not kill adults and have not changed over 20 years. There is a concern about resistance and a need for new antifilarial drugs (Kumar *et al.*, 2007). Although levamisole has been used for treatment (Mak & Zaman, 1980), it requires multiple doses. Development of novel cholinergic anthelmintics (derquantel, monepantel and the combination derquantel+avermectin) for other nematode parasites makes evaluation and characterization of *Brugia* nicotinic acetylcholine receptor channels (nAChRs) as target sites timely and imperative. Recent publication of the draft genome sequence of *B. malayi* has allowed identification of the nAChR subunit genes (Williamson *et al.*, 2007), and encourages the characterization of native receptors.

Anthelmintic resistance that follows mass drug administration (MDA) creates an urgent need for basic research and identification of new drugs. For MDA, it is easier to administer the therapeutic agent as a single dose without adjusting for weight. Albendazole is easier to administer for this reason and is used more widely. Fewer countries use levamisole and pyrantel on a wide scale because of the need to adjust for weight of individuals. The increased albendazole/mebendazole use appears to be leading to development of resistance in STNs of humans (Albonico *et al.*, 2005) as it did in animals (Jackson, 1993; Kaplan, 2004). To address this problem of benzimidazole resistance, additional drugs are required, with a different site of action and suitable for prophylactic mass treatment with easy administration. Tribendimidine has been suggested as one drug for STN single-dose MDA. In filaria, there is also evidence of the emergence of resistance because of the regular use of ivermectin (Osei-Atweneboana *et al.*, 2007). It is widely recognized that existing single-dose MDAs are limited and there is an urgent need to support research that allows development of novel anthelmintics or *combinations* that increase spectrum and limit resistance (Leathwick, 2013).

## Cross-resistance

Development of resistance to anthelmintics has followed continuous use for prophylaxis in animals. The resistance to one drug in a particular drug class has been associated with resistance to other drugs in that same class (Sangster & Gill, 1999). This is referred to as *cross-resistance* and is seen for drugs in the benzimidazole (albendazole could show cross-resistance with fenbendazole) and avermectin (ivermectin could show cross-resistance with doramectin) classes.

## Nematode nicotinic receptors and anthelmintics

**Fig. 3:**
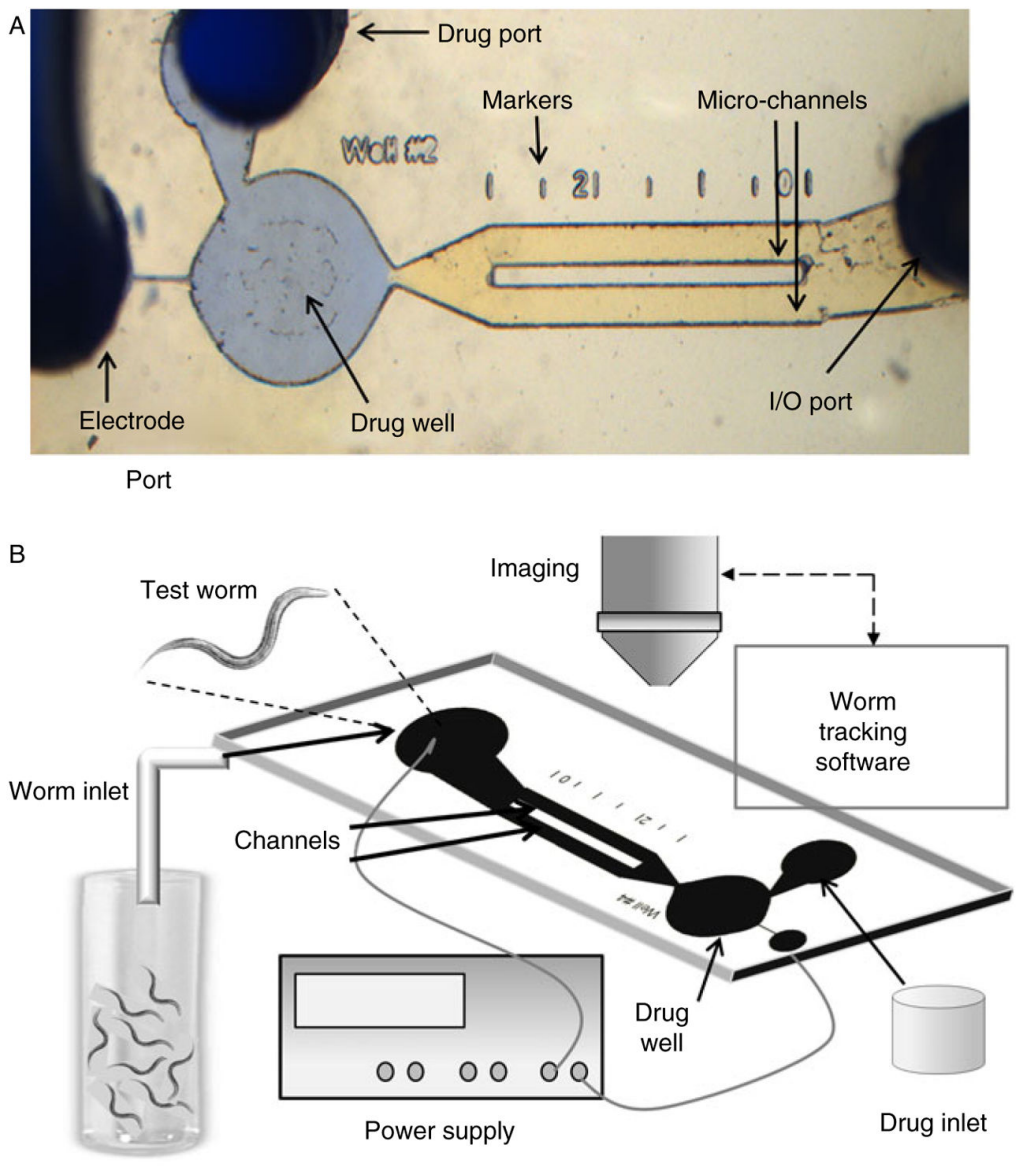
(colour online) Microfluidic chamber with drug well and micro-channels. The L3 larvae of nematode parasites or adult *Caenorhabditis elegans* are placed in the input–output port (worm inlet 1/0 port) via syringe and catheter. They can be driven into the drug well and held under a voltage field, and released at a defined time by reversing the voltage. The escape and behaviour of the larvae/worms following entry into the drug field is tested at different drug concentrations. The swimming of the larvae/worms in the channels is described by a sinusoidal wave with measurement of frequency (*N*), wavelength (*λ*), amplitude (*a*) and velocity (*v*).

Major classes of anthelmintic act on ligand-gated ion-channels (LGICs), demonstrating that these channels are validated drug target sites, which should be characterized more fully to facilitate novel drug development and use. The nematode parasite neuromuscular system has provided a good drug target system for anthelmintics. We can now monitor the effects of drugs that target the neuromuscular system by measuring precise changes in the motility of the parasites. New microfluidic techniques (fig. 3) allow drug effects on the neuromuscular system to be dissected and quantitated through recording and analysing the sinusoidal movement of L3 larvae, using measurements of the amplitude (*a*), frequency (*N*), wavelength (*λ*) and velocity (*v*) to observe effects of the anthelmintics on these parameters (Chen *et al.*, 2011). It has also been found that the nematode L3 larvae are sensitive to applied electrical fields and can be corralled into a drug well or driven out of the drug well at fixed times to measure anthelmintic drug effects on the neuromuscular system (Carr *et al.*, 2011). It is likely that the microfluidic technology will allow better characterization of different phenotypes or isolates of the same species of nematode parasite: anthelmintic-sensitive and anthelmintic-resistant isolates differ in their motility (Carr *et al.*, 2011; Chen *et al.,* 2011).

Pyrantel and levamisole are selective agonists on nematode nAChRs. Ivermectin and moxidectin are allosteric modulators of channels that include the glutamate-gated chloride channels and nAChRs. We can see that LGICs are validated target sites by being sites of action of existing anthelmintics. LGICs are also good target sites for novel drugs, provided the new drugs are not made redundant by cross-resistance. The focus of this article is on cholinergic drugs that activate selectively the nAChRs.

Application of modern molecular methods based on small ribosomal DNA subunit sequences (Blaxter *et al.*, 1998; Holterman *et al.*, 2006) divide the phylum Nematoda into Clades I, II, III, IV and V. *Caenorhabditis elegans* is in Clade V and has been a helpful genetic model for Clade V (Rhabditidae) parasitic nematodes, such as *O. dentatum*. Its genome has been sequenced and the nAChR subunit genes have been identified. However, accumulating evidence suggests that *C. elegans* is limited as a model for parasitic nematodes. *Caenorhabditis elegans* appears to have the largest number of nAChR subunit genes of all nematodes in contrast to those of Clade III, which are phylogenetically distant, and which have far fewer nAChR subunit genes (Williamson *et al.*, 2007). In *C. elegans*, the subunit genes *unc-38*, *unc-29*, *unc-63*, *lev-1* and *lev-8* are required to encode for the levamisole nAChR but *lev-1* appears to be missing in Clade III parasites such as *B. malayi* and *A. suum*.

**Fig. 4:**
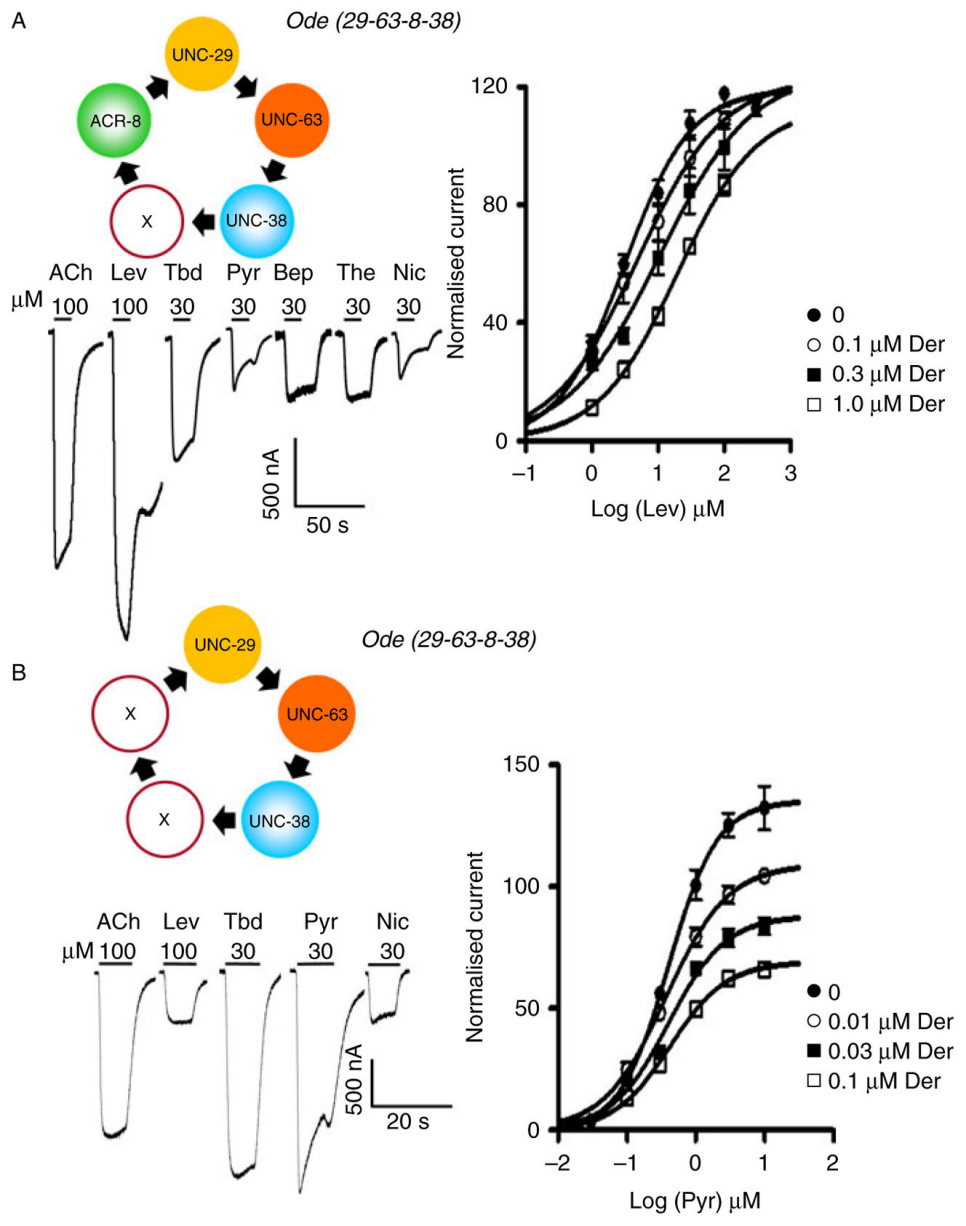
(colour online) Expression of different parasite nAChRs using *Xenopus* oocyte expression reveals the effect of subunit combinations on anthelmintic sensitivity. Antagonism of derquantel depends on nAChR subtype, demonstrated using two of the four different expressed *O. dentatum* subunits that can occur. (A) Expression of Ode-UNC-63:Ode-UNC-29:Ode-UNC-38:Ode-ACR-8 in *Xenopus* oocytes produces receptors that, under voltage clamp, produce currents to acetylcholine, levamisole, tribendimidine, pyrantel, bephenium, thenium and nicotine, with the biggest current to *levamisole*. This receptor is *competitively* antagonized by derquantel (plot). (B) Expression of Ode-UNC-63:Ode-UNC-29:Ode-UNC-38 in *Xenopus* oocytes produces receptors that, under voltage clamp, produce currents to acetylcholine, levamisole, tribendimidine, pyrantel, bephenium, thenium and nicotine, with the biggest current to *pyrantel*. This receptor is *non-competitively* antagonized by derquantel (plot).

Knowledge has advanced of genomes of *B. malayi* (Ghedin *et al.*, 2007), *A. suum* (Jex *et al.*, 2011) and transcriptomes of *O. dentatum* (Romine *et al.*, 2013). With new sequencing technology, knowledge of the genomes of representative parasitic nematodes has advanced rapidly with *B. malayi* and *A. suum*. However, compared to *C. elegans,* there is a bottleneck; there are a limited number of functional studies from parasitic nematodes characterizing ion-channels of neuromuscular transmission. Additional functional studies of ion-channels in native preparations of parasitic nematodes or expressed ion-channels are required. There are only a limited number of *in vivo* reports of ion-channels from filaria. The Saz group described contraction and electrophysiological studies from the filarid *Dipetalonema vitiae* body muscle and reported responses to acetylcholine (Rohrer *et al.*, 1988; Christ *et al.*, 1990, 1994). Cloned and expressed glutamate-gated chloride channels from *Dirofilaria immitis* have demonstrated ivermectin sensitivity (Yates & Wolstenholme, 2004). For *A. suum*, *H. contortus* and *O. dentatum,* the situation is better; there are observations from *A. suum* body muscle (Martin, 1982, 1985; Robertson & Martin, 1993; Evans & Martin, 1996; Qian *et al.*, 2006) and *A. suum* expressed channels (Williamson *et al.*, 2009; Bennett *et al.*, 2012); *H. contortus* expressed receptors (Boulin *et al.,* 2011) and *O. dentatum* body muscle (Robertson *et al.*, 1999; Qian *et al.*, 2006) and *O. dentatum* expressed channels (Buxton *et al.*, 2014). One of the interesting and significant findings is that the subunit combination of the nAChR receptor affects the sensitivity to the nicotinic anthelmintic drug (Williamson *et al.*, 2009; Buxton *et al.*, 2014). Figure 4 illustrates expression of two (of the four) nAChR subtypes that have been expressed in *Xenopus* oocytes using combinations of muscle subunit cRNAs of *Ode-unc-63, Ode-unc-38, Ode-unc-29* or *Ode-acr-8* (Buxton *et al.*, 2014). The Ode-UNC-63:Ode-UNC-29:Ode-UNC-38:Ode-ACR-8 subtype is most sensitive to levamisole and is competitively antagonized by derquantel, but Ode-UNC-63:Ode-UNC-29:Ode-UNC-38 is more sensitive to pyrantel and is non-competitively antagonized by derquantel. Interestingly, the Ode-UNC-63:Ode-UNC-29:Ode-UNC-38:Ode-ACR-8, has a 35 pS single channel conductance like the *in vivo* L-subtype receptor that is lost with levamisole resistance (Robertson *et al.*, 1999), suggesting that it corresponds to the native receptor. The different subtypes of nAChR present *in vivo* (Robertson & Martin, 1993; Qian *et al.*, 2006) and the expression experiments (Williamson *et al.*, 2009; Buxton *et al.*, 2014) suggest that the cholinergic anthelmintic receptors may be more variable (and plastic) than we had anticipated. We do not know yet if, or how, this variability may play a role in resistance to the cholinergic anthelmintics.

**Fig. 5:**
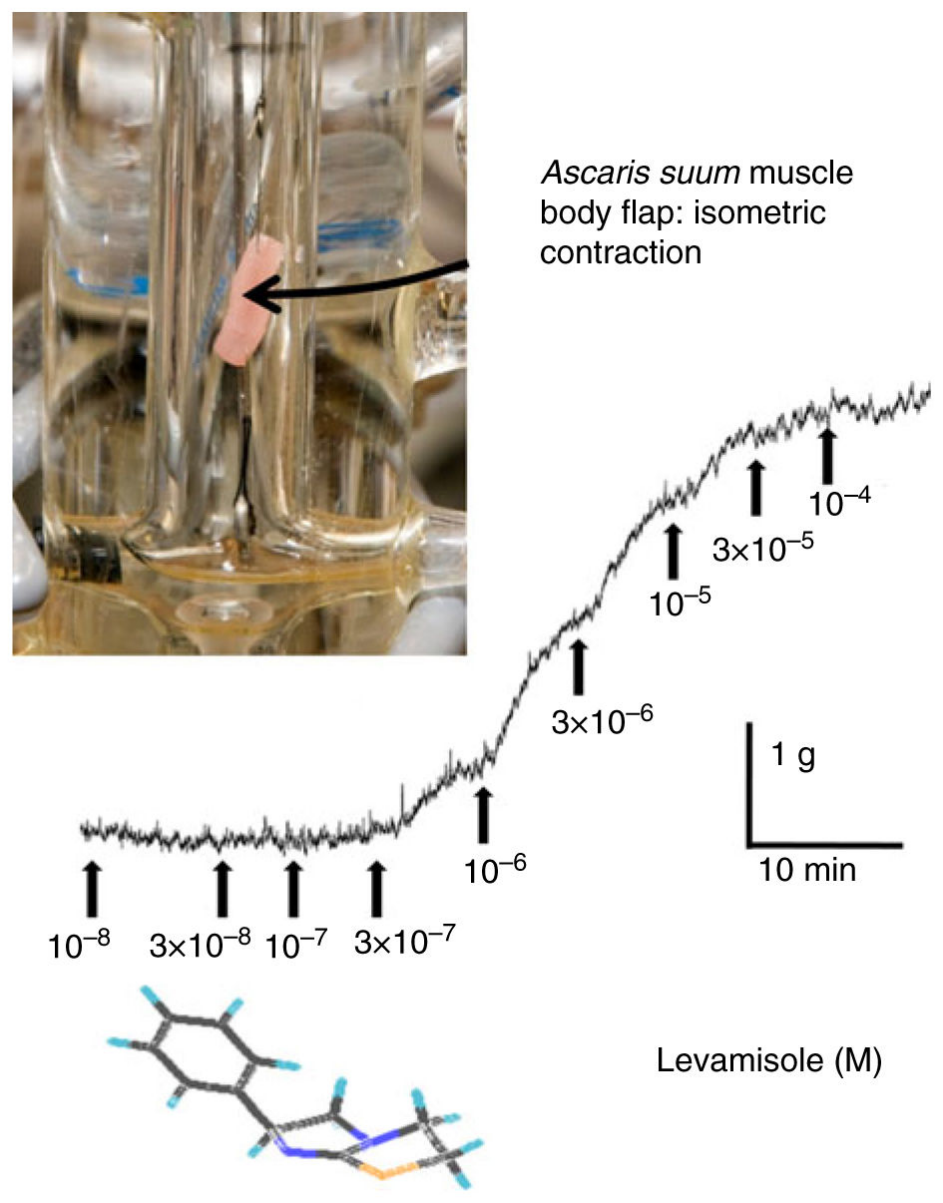
(colour online) Photograph of an *Ascaris* body muscle flap being tested with levamisole under isometric contraction. The trace shows the contraction effect of levamisole applied in cumulative doses.

Nicotinic anthelmintics such as pyrantel and levamisole selectively paralyse nematodes by activating cholinergic ion-channels (nAChRs) in their body muscle to produce contraction and spastic paralysis. Figure 5 illustrates the effect of cumulative application of levamisole to a muscle strip of *A. suum.* The importance of nematode nAChRs as drug targets has increased because of the recent introduction of novel cholinergic compounds. The new cholinergic anthelmintics, morantel tribendimidine and derquantel (Kaminsky *et al.*, 2008; Rufener *et al.*, 2009; Buxton *et al.*, 2014) have different nAChR subtype selectivities. Figure 4, for example, shows that tribendimidine is more potent than levamisole on the *Xenopus* oocyte expressed Ode(29-63-38) subtype, and that levamisole is more potent than tribendimidine on the Ode(29-63-8-38) subtype. The presence *in vivo* of different cholinergic receptor subtypes activated by anthelmintics has been demonstrated in experiments using the *A. suum* muscle contraction strip (fig. 5) and the antagonists derquantel, paraherquamide and methyllycaconitine (MLA). Paraherquamide is a more potent antagonist of nicotine than bephenium, and nicotine and bephenium are selective for different receptor subtypes. The cholinergic anthelmintic subtypes are separated into at least three *in vivo* subtypes: N-, L- and B-subtypes ((Robertson *et al.*, 2002; Qian *et al.*, 2006). The three different subtypes present in *A. suum* are: the N-subtype, which is nicotine selective; the L-subtype, which is levamisole selective; and the B-subtype, which is bephenium selective; suggesting that resistance to the anthelmintic which stimulates the L-subtype may not be associated with resistance to anthelmintics which stimulate the N-subtype or the B-subtype.

Few drugs with optimal properties exist and only albendazole is usually used for single-dose MDA. Tribendimidine has been developed by the Chinese Centers for Disease Control and Prevention and has potential for single-dose MDA, but details of its action in parasites are not clear. It is a promising anthelmintic with a symmetrical diamidine structure. The China State Food and Drug Administration approved it for human use in 2004 (Steinmann *et al.*, 2008). It has broad-spectrum action in single-dose therapy against parasitic nematodes of humans, with effects against *Ascaris*, hookworm and *Strongyloides* (Xiao *et al.*, 2005). There is, however, some concern about the rapid metabolism of tribendimidine and production of potential carcinogens.

Little was known of the mode of action of tribendimidine until Hu *et al.* (2009) published observations on *C. elegans.* These authors used molecular experiments to suggest that the site of action of tribendimidine was the levamisole receptor (nAChR). These experiments are helpful but the observations are limited because the levamisole receptor of *C. elegans* has different pharmacological properties to the muscle receptors of parasitic nematodes. The Clade V *C. elegans* levamisole-sensitive receptor has different subunits and is not activated by nicotine. Observations on *O. dentatum* nAChR expressed in *Xenopus* oocytes show that tribendimidine has a different subtype selectivity to levamisole (Buxton *et al.*, 2014) and may not, therefore, show cross-resistance with levamisole.

## Derquantel and abamectin

Paraherquamide-A and macfortine-A are fermentation products of a *Penicillium* species that were discovered by Merck. Subsequently, Pfizer used nematode parasites that were resistant to levamisole, benzimidazoles and macrocyclic lactones, and maintained in jirds, to look for compounds that were ‘resistance busting’. The medicinal chemists modified the structure of paraherquamide by reducing the C-ring ketone of paraherquamide-A, to produce a less host-toxic 2-desoxyparaherquamide derivative which was given the generic name, derquantel. Derquantel is equally effective when given orally, intravenously, intraperitoneally or intramuscularly (Johnson *et al.*, 2004) in jirds against gastrointestinal levamisole-resistant parasites. It has been introduced as an animal anthelmintic with an avermectin, abamectin, because clinically they have additive/synergistic effects (Little *et al.,* 2010).

We identified the mode of action of derquantel in muscle strip preparations of *A. suum* and found that derquantel was a competitive, but selective, cholinergic antagonist (Robertson *et al.*, 2002). Parasitic gastrointestinal nematodes resistant to levamisole retain sensitivity to the B-subtype agonist, bephenium (Sangster *et al.*, 1991) and derquantel has B-subtype selectivity (Qian *et al.*, 2006). These observations may explain why derquantel does not show cross-resistance to levamisole and why it has ‘resistance busting’ properties.

**Fig. 6:**
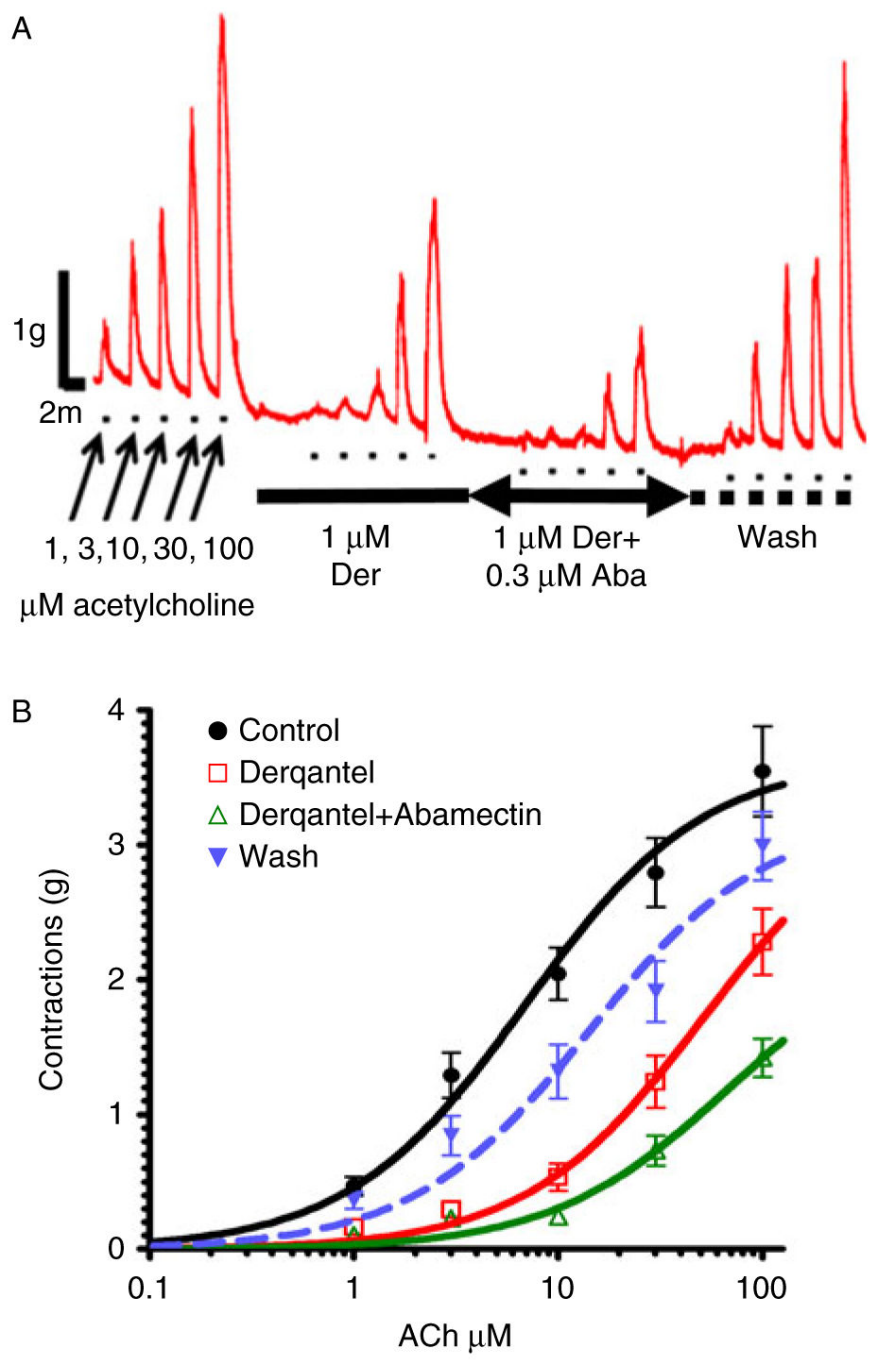
(colour online) (A) Isometric contraction of *Ascaris suum* muscle strips produced by application of increasing concentrations of acetylcholine and antagonism by 1 μM derquantel, 1 μM derquantel + 0.3 μM abamectin and wash. Note that derquantel decreases the responses to acetylcholine and that the addition of abamectin increases the inhibition. (B) The concentration-depolarizing–response plot of acetylcholine, showing mean ± standard error (SE) bars. Control (*n* = 11, filled circles); in the presence of 1 μM derquantel (*n* = 11); 1 μM derquantel + 0.3 μM abamectin. The predicted additive effect is shown by the dashed line. The derquantel–abamectin combination is statistically (*P* < 0.05) more inhibitory than additive at concentrations > 10 μM acetylcholine.

A significant observation (Puttachary *et al.*, 2013) that we have made using *A. suum* muscle in our muscle contraction assay and, separately, in current-clamp experiments, is that derquantel interacts additively with the macrocyclic lactone, abamectin (fig. 6). At higher acetylcholine concentrations the interaction is synergistic. This is a novel and important observation and suggests that the combination approach of derquantel + abamectin can enhance the therapeutic effects of the anthelmintics.

## *Brugia malayi* preparations

**Fig. 7:**
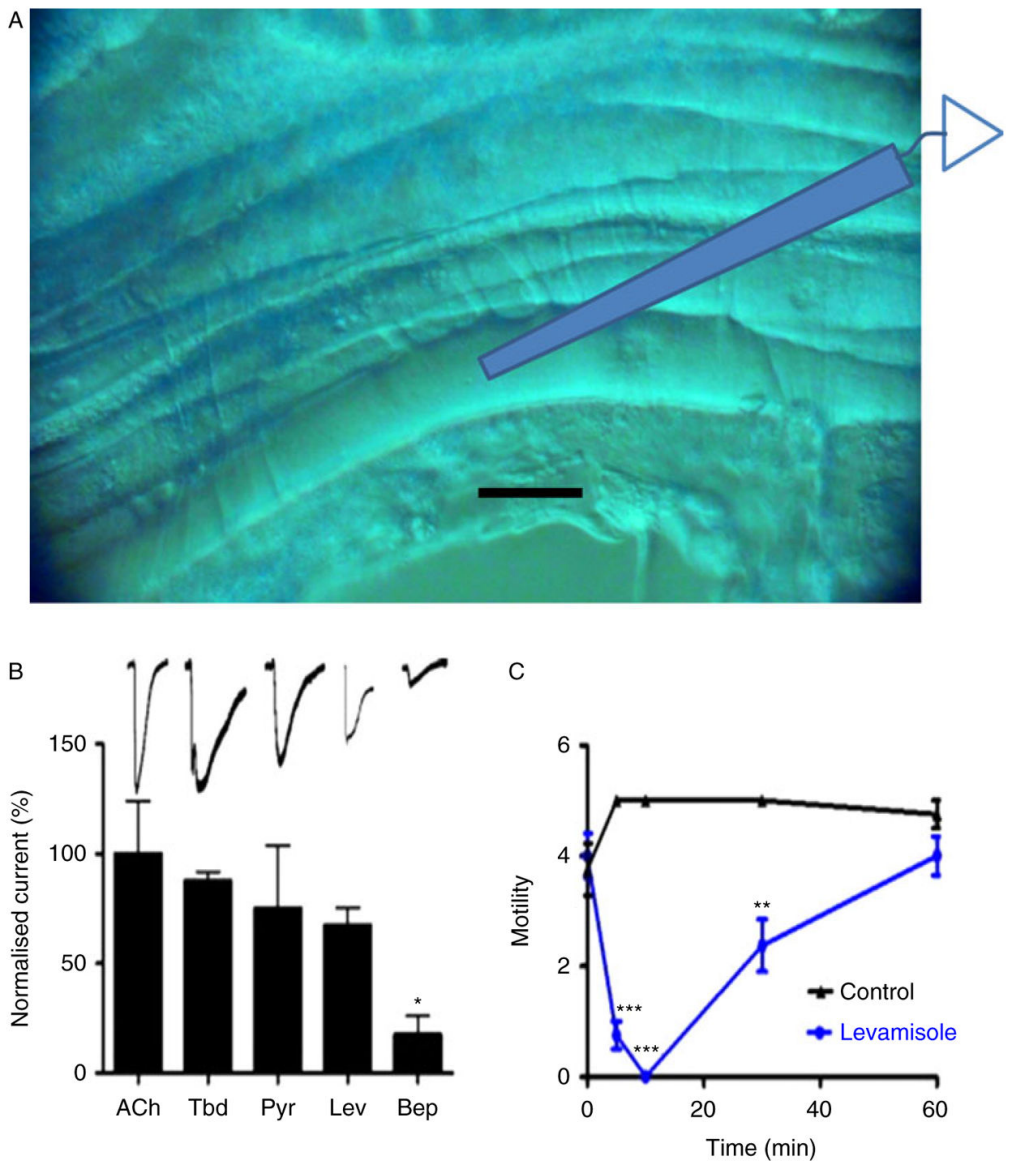
(colour online) Nomarski photomicrograph of a *Brugia malayi* muscle flap dissection with a diagram of a patch-pipette placed for whole-cell current clamp recording of the nicotinic receptors. Scale bar 10 μm. (B) Bar chart (mean ± SE) of normalized currents produced by the nAChR agonists/anthelmintics (30 μM) on *B. malayi* muscles in whole-cell patch-clamp. All current responses were normalized to ACh currents (*P < 0.05, paired *t*-test). Displayed above the bars are sample whole-cell current traces. Scale bar, horizontal 30 s, vertical 700 pA. (C) Plot of motility versus time (min) of adult female *B. malayi* in the absence and presence of 30 μM levamisole. Four worms/treatment were used for the assay. Comparisons of motility were made between control and treated worms at each time point, **P < 0.01, ***P < 0.001.

We are particularly excited by developments of the *B. malayi* muscle-flap preparation for recording from single muscle cells (Robertson *et al.*, 2011, 2013). We glue one side of a 0.5 cm body cylinder section with GluShield (^®^Glushield, Seattle, Washington, USA) delivered with a modified patch pipette under the high power of the dissecting microscope. The body cylinder is then cut open and, after removal of the intestine and uterus, it is glued flat. We use Nomarski optics (fig. 7) to view the muscle cells. We make patch recordings following brief collagenase treatment to clear the extracellular matrix. Single-channel recordings using cell-attached and isolated inside-out patch recordings or whole-cell recordings allow us to study nAChRs in the intact preparation. Figure 7B shows our recently developed recording system for whole-cell currents made from a *B. malayi* muscle (Robertson *et al.*, 2011, 2013). It shows the sequential application of 30 μM acetylcholine, tribendimidine, pyrantel, levamisole and bephenium along with a bar chart of normalized currents. Interestingly bephenium is the least potent of the agonist series, which is different from the situation in *A. suum* muscle, suggesting that the receptor subtypes present are different from the receptors of *B. malayi.* Figure 7C shows the effect of longer-term application of 30 μM levamisole on adult motility, and that after a period of some 10 min the inhibition of motility begins to be lost.

## Concluding comments

The neglected tropical diseases, produced by phylogenetically separate Clades of nematode parasites, produce widespread and debilitating diseases in humans (de Silva *et al.*, 2003; Bethony *et al.*, 2006; Hotez *et al.*, 2008). Anthelmintic drugs are used for treatment and prophylaxis but there are concerns about the development of resistance in humans (Diawara *et al.*, 2009, 2013) as in animals (Kaplan, 2004). There is a need for effective drugs against soil-transmitted nematodes (Hu *et al.*, 2013) and filaria (Prichard, 2005). We think that the introduction of the new microfluidic technology and expression systems and research on the ligand-gated ion-channels is timely, specifically the nicotinic acetylcholine receptors, and therapeutically relevant. There is a need to use molecular and functional assays to study similarities, pharmacological diversity and modulation of the nAChR subtypes in the different Clades. The subtypes appear as separate target sites for the classic anthelmintics, monepantel and derquantel. Knowledge of subtype selectivities and drug interactions (e.g. derquantel and avermectins) allows combinations of anthelmintics to be produced to cover broader spectra of receptors and parasites. These are anticipated to be more effective therapeutically in the face of resistance. The need for other novel ‘resistance busting’ anthelmintics from other classes of anthelmintic exists for human as well as animal nematode parasite control. There is still an urgent need for vaccines, antivectoral agents, improved sanitation, better diagnostic methods and markers for resistance, while combinations of anthelmintics will also need to be studied at the basic and clinical levels. There are many interesting and exciting basic science and translational issues that need to be addressed. We look forward to seeing the field develop over the coming years.
